# Multicenter phase II study of capecitabine plus oxaliplatin in older patients with advanced gastric cancer: the Tokyo Cooperative Oncology Group (TCOG) GI-1601 study

**DOI:** 10.1007/s10120-023-01423-z

**Published:** 2023-08-23

**Authors:** Ryohei Kawabata, Keisho Chin, Daisuke Takahari, Hisashi Hosaka, Osamu Muto, Yoshiaki Shindo, Naoki Nagata, Hiroshi Yabusaki, Hiroshi Imamura, Shunji Endo, Tomomi Kashiwada, Masato Nakamura, Jun Hihara, Michiya Kobayashi, Tamotsu Sagawa, Soh Saito, Atsushi Sato, Takeshi Yamada, Naohiro Okano, Ken Shimada, Masashi Matsushima, Masato Kataoka, Shigemi Matsumoto, Masahiro Goto, Masahito Kotaka, Takeshi Shiraishi, Hiromichi Yamai, Fumio Nagashima, Naoki Ishizuka, Kensei Yamaguchi

**Affiliations:** 1 Department of Surgery, Osaka Rosai Hospital, Nagasone-cho, Kita-ku, Sakai, Osaka 1179-35918025 Japan; 2 Department of Gastroenterological Chemotherapy, Cancer Institute Hospital of the Japanese Foundation for Cancer Research, Tokyo, Japan; 3 Department of Gastroenterology, Gunma Prefectural Cancer Center, Gunma, Japan; 4 Department of Medical Oncology, Japanese Red Cross Akita Hospital, Akita, Japan; 5 Department of Gastroenterological Surgery, Nakadori General Hospital, Akita, Japan; 6 Department of Gastroenterological Surgery, Kitakyushu General Hospital, Kitakyushu, Fukuoka Japan; 7 Department of Gastroenterological Surgery, Niigata Cancer Center Hospital, Niigata, Japan; 8 Department of Gastroenterological Surgery, Toyonaka Municipal Hospital, Toyonaka, Osaka Japan; 9 Department of Gastroenterological Surgery, Yao Municipal Hospital, Yao, Osaka Japan; 10 Department of Internal Medicine, Division of Hematology, Respiratory Medicine and Oncology, Faculty of Medicine, Saga University, Saga, Japan; 11 Aizawa Comprehensive Cancer Center, Aizawa Hospital, Matsumoto, Nagano Japan; 12 Department of Gastroenterological Surgery, Hiroshima City North Medical Center Asa Citizens Hospital, Hiroshima, Japan; 13 Cancer Treatment Center, Kochi Medical School Hospital, Nankoku, Kochi Japan; 14 Department of Gastroenterology, National Hospital Organization Hokkaido Cancer Center, Sapporo, Hokkaido Japan; 15 Department of Gastroenterology, Misawa Citi Hospital, Misawa, Aomori Japan; 16 Department of Medical Oncology, Hirosaki University Graduate School of Medicine, Hirosaki, Aomori Japan; 17 Department of Gastroenterology, Faculty of Medicine, University of Tsukuba, Tsukuba, Ibaragi Japan; 18 Department of Medical Oncology, Kyorin University Faculty of Medicine, Mitaka, Tokyo Japan; 19 Department of Internal Medicine, Division of Medical Oncology, Showa University Koto-Toyosu Hospital, Tokyo, Japan; 20 Department of Gastroenterology, Tokai University School of Medicine, Tokyo, Japan; 21 Department of Surgery, National Hospital Organization Nagoya Medical Center, Tokyo, Japan; 22 Department of Clinical Oncology, Kyoto University Hospital, Kyoto, Japan; 23 Cancer Chemotherapy Center, Osaka Medical and Pharmaceutical University Hospital, Suita, Osaka Japan; 24 Gastrointestinal Cancer Center, Sano Hospital, Tokyo, Japan; 25 Department of Medical Oncology, Japanese Red Cross Matsuyama Hospital, Matsuyama, Ehime Japan; 26 Department of Gastroenterological Surgery, Japanese Red Cross Kochi Hospital, Kochi, Japan; 27 Clinical Planning and Strategy Department Center for Development of Advanced Cancer Therapy, Cancer Institute Hospital of the Japanese Foundation for Cancer Research, Tokyo, Japan

**Keywords:** Gastric cancer, Older patients, Capecitabine, Oxaliplatin

## Abstract

**Background:**

Capecitabine plus oxaliplatin **(**CapeOX) is a standard treatment option for advanced gastric cancer (AGC). We conducted a prospective multicenter phase II study to evaluate the efficacy and safety of CapeOX as a first-line therapy for AGC in older patients.

**Methods:**

Chemotherapy-naive patients aged ≥ 70 years with AGC were eligible. Initial treatment comprised capecitabine (2000 mg/m^2^ on days 1–14) and oxaliplatin (130 mg/m^2^ on day 1) every 3 weeks. After the initial feasibility assessment, the dose was reduced considering toxicity (capecitabine, 1500 mg/m^2^ on days 1–14; and oxaliplatin, 100 mg/m^2^ on day 1 every 3 weeks). The primary endpoint was overall survival (OS).

**Results:**

In total, 108 patients were enrolled, of whom 104 were evaluated. Thirty-nine patients received the original-dose treatment, whereas 65 received the reduced-dose treatment. The median OS, progression-free survival (PFS), and time to treatment failure (TTF) were 12.9 (95% CI 11.6–14.8), 5.7 (95% CI 5.0–7.0), and 4.3 (95% CI 3.9–5.7) months, respectively, for all patients; 13.4 (95% CI 9.5–16.0), 5.8 (95% CI 4.1–7.8), and 5.3 (95% CI 3.5–7.2) months in the original-dose group; and 12.8 (95% CI 11.3–15.3), 5.7 (95% CI 4.4–7.0), and 4.1 (95% CI 3.7–5.7) months in the reduced-dose group. The most common grade 3/4 toxicities were neutropenia (17.9%), anemia (12.8%), and thrombocytopenia (12.8%) in the original-dose group and neutropenia (13.8%) and anorexia (12.3%) in the reduced-dose group.

**Conclusions:**

These findings demonstrate CapeOX's efficacy and safety in older AGC patients.

## Introduction

Gastric cancer is the third leading cause of cancer‐related death and the fifth most common malignancy diagnosed worldwide [1]. The standard treatment for advanced gastric cancer (AGC) is chemotherapy, which is given to delay the manifestation of, or ameliorate, symptoms and prolong survival [2]. Recently, older patients have comprised the greatest proportion of patients diagnosed with gastric cancer, with 77% of patients diagnosed over the age of 75 years [3, 4]. This patient population faces unique challenges, including short life expectancy, multiple comorbidities, multi-drug use, physiological decline (aging phenomenon), malnutrition, cognitive function limitations, and socioeconomic limitations [5]. As such, individualized treatment strategies are required for older patients.

Based on the results of a previous randomized controlled trial, the sixth edition of the Japanese Gastric Cancer Treatment Guidelines defines a regimen combining a platinum-based agent and a fluorouracil-based agent, including S-1 plus oxaliplatin (SOX), capecitabine plus oxaliplatin (CapeOX), and FOLFOX, as an acceptable first-line chemotherapy regimen for patients with epidermal growth factor receptor 2 (HER2)-negative AGC [2]. However, most participants in these trials were younger patients. Few reports provide a high level of evidence regarding effective chemotherapeutic modalities against AGC in older patients [6–9]. While some reports show that combinatorial therapy comprising a fluoropyrimidine drug and a platinum drug is preferable, others suggest that a fluoropyrimidine drug alone might have the same effect. Hence, no consensus has been reached regarding optimal chemotherapy regimens for older patients [10–14].

Capecitabine is an oral fluoropyrimidine rationally designed to preferentially generate 5-fluorouracil (5-FU) in tumors and might be a safer option than S-1 in older patients [15]. Although studies evaluating CapeOX in older patients have been reported, data for the Japanese population are lacking [16–19]. In the therapeutic development of cytotoxic anticancer agents, dose-limiting toxicity, the maximum tolerated dose, and the recommended dose often differ among ethnic groups; thus, care must be taken when extrapolating clinical data across geographical regions.

Therefore, this prospective multicenter phase II study (UMIN000022450/jRCTs051180126) aims to examine the efficacy and safety of CapeOX in Japanese older patients with AGC.

## Methods

### Patients

The Tokyo Cooperative Oncology Group (TCOG) GI-1601 study is a multicenter, prospective, phase II trial to examine the efficacy and safety of CapeOX in Japanese older patients with AGC. The inclusion criteria comprised: histologically diagnosed gastric adenocarcinoma and HER2-negative cancer or cancer of unknown cause; unresectable, metastatic, or recurrent disease; adequate self-supported nutritional intake; age ≥ 70 years; Eastern Cooperative Oncology Group (ECOG) performance status of 0 or 1; no history of chemo- or radiotherapy; adequate organ function as defined by a hemoglobin level ≥ 8 g/dL, white blood cell count ≤ 12,000/mm^3^, absolute neutrophil count ≥ 1,500/mm^3^, platelet count ≥ 100,000/mm^3^, total bilirubin level ≤ 1.5 mg/dL, serum transaminase ≤ 100 IU/L, serum creatinine ≤ 1.5 mg/dL, and creatinine clearance ≥ 50 mL/min; evaluable lesion according to the Response Evaluation Criteria In Solid Tumors (RECIST) version 1.1 [20]. The exclusion criteria comprised: active bleeding from the main tumor; moderate or severe ascites; peripheral neuropathy, brain metastasis, or active coexisting cancer.

All patients provided written informed consent before enrollment. The study was performed in accordance with the Declaration of Helsinki and Ethical Guidelines for Clinical Studies in Japan and was approved by the ethics committees of TCOG and each participating center before initiating enrollment. An Independent Data Monitoring Committee (IDMC) reviewed all efficacy and safety protocols. This study was registered in UMIN-CTR under code UMIN000022450 and in the Japan Registry of Clinical Trials under code jRCTs051180126.

### Treatment plan

The original-dose CapeOX treatment comprised capecitabine (1,000 mg/m^2^ bis in die [b.i.d.] on days 1–14 of each course) and oxaliplatin (130 mg/m^2^ intravenously on day 1 of each course) every 3 weeks. As described by Chin et al*.*, safety analyses were performed upon completion of the initial two courses of chemotherapy for the first 20 patients; the IDMC considered the tolerability of chemotherapy from the initial safety data, according to the protocol [21]. The applied criterion included ≥ 30% (6/20) of patients requiring two levels of dose reduction due to an adverse event, in addition to suspension or cessation of chemotherapy. If CapeOX was deemed intolerable, the trial would be continued with dose modification.

Five patients were unable to complete the initial two courses of chemotherapy due to toxicity, necessitating dose reduction (10/15) or suspension of the subsequent course (12/15). Considering these results, the IDMC recommended continuing the study with a reduced-dose CapeOX treatment comprising capecitabine (750 mg/m^2^, b.i.d., on days 1–14 of each course) and oxaliplatin (100 mg/m^2^ intravenously on day 1 of each course) every 3 weeks. Additionally, prophylactic antiemetic therapy for oxaliplatin was administered. Treatment delays, suspensions, or dose modifications were performed according to the J-CLASSIC study [22]. Capecitabine was also suspended in the event of any ≥ grade 3 hematological toxicity or ≥ grade 2 non-hematological toxicity, excluding grade 2 nausea or vomiting judged to be related to capecitabine; treatment was resumed once the toxicity had resolved to grade ≤ 1 at a capecitabine dose that was reduced to 75% of the starting dose. In cases of a second episode of ≥ grade 3 hematological toxicity or ≥ grade 2 non-hematological toxicity, the dose was reduced to 50% of the starting dose. Meanwhile, after the occurrence of a single grade 3 toxicity event, the oxaliplatin dose in the subsequent course was reduced to 100 mg/m^2^ in the original-dose group and 85 mg/m^2^ in the reduced-dose group. Oxaliplatin and capecitabine were discontinued in the event of grade ≥ 4 non-hematological toxicity. If an oxaliplatin-related allergic reaction or peripheral neuropathy occurred, capecitabine could be continued as monotherapy.

### Evaluations

The primary endpoint was overall survival (OS). The secondary endpoints were progression-free survival (PFS), time to treatment failure (TTF), response rate, and adverse events. Tumors were assessed every 8 weeks until disease progression, and objective responses were evaluated according to RECIST version 1.1 [20]. OS was defined as the time from registration until death from any cause and was censored at the time of the last visit for patients who were lost to follow-up. PFS was defined as the time from registration until objective tumor progression or death from any cause. TTF was defined as the time from registration to treatment discontinuation or last follow-up. Adverse events were evaluated according to the National Cancer Institute Common Terminology Criteria for Adverse Events (NCI-CTCAE) version 4.03 [23].

### Statistical analysis

Our trial was designed as a therapeutic exploratory study to compare our CapeOX therapy with historical reference data in the first-line treatment of older patients with AGC. However, at the time of the trial’s planning, historical reference data for older gastric cancer were scarce. Koizumi and Imamura reported the results of S-1 monotherapy in older patients with AGC, with a median OS of 10.4 months and 9.2 months, respectively [10, 11]. In addition, an interim analysis of a phase III trial conducted by Hwang et al*.* reported that the median OS time for CapeOX therapy was 13.5 months [18]. Hence, in the current study, the required sample size was calculated as 105 patients based on a threshold median survival time (MST) of 10 months, an expected MST of 13.5 months, a power of 80%, a two-sided α value of 5%, an accrual period of 3 years, and a follow-up period of 1.5 years.

The primary analysis was based on the full analysis set (FAS), comprising all enrolled patients excluding those deemed ineligible after enrollment. We used the Kaplan–Meier method to estimate survival curves and Greenwood’s formula to calculate 95% confidence intervals (CIs) for survival rates. Log-rank test was applied to compare survival curves. Statistical analyses were conducted using SAS version 9.4 (SAS Institute, Cary, NC).

## Results

### Patient characteristics

**Fig. 1:**
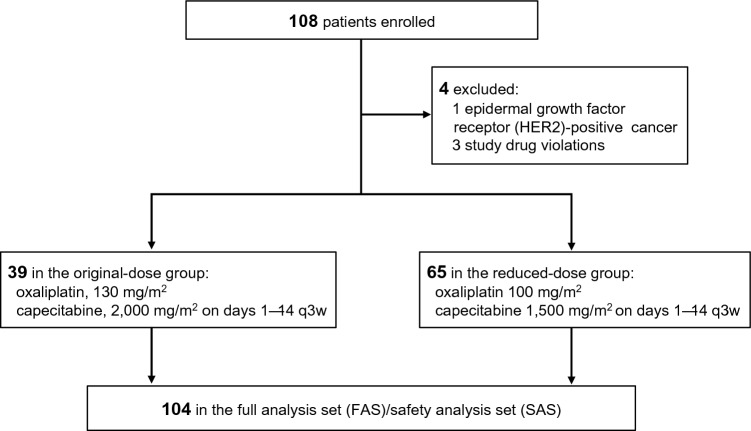
CONSORT diagram

Patients were enrolled from September 2016 to June 2020. After enrolling the initial 39 patients treated with original-dose CapeOX (original-dose group), the protocol was revised to incorporate IDMC recommendations, and the subsequent 69 patients were treated with reduced-dose CapeOX (reduced-dose group). The last follow-up analysis was conducted in July 2022. Finally, 108 patients from 26 centers in Japan were enrolled in this study. However, one patient with HER2-positive cancer and three patients with study drug violations—administered generic drugs—were excluded. A total of 104 patients were included in the FAS (Fig. 1), the demographic data is presented in Table 1. The median age was 76 (range 70–88) years, and the ECOG performance status was 0 in 66 patients and 1 in 38 patients. Fifty-two patients (50%) had differentiated adenocarcinoma, 83 (79.8%) had unresectable lesions, and 21 (20.2%) had recurrent disease. The most frequent site of metastasis was the lymph nodes (55.8%), followed by the liver (33.7%), peritoneum (33.7%), and lungs (8.7%).

**Table 1:** Patient characteristics

	All (*n* = 104)	Original dose (*n* = 39)	Reduce dose (*n* = 65)
*Sex*			
Male	67 (64.4%)	26 (66.7%)	41 (63.1%)
Female	37 (35.6%)	13 (33.3%)	24 (36.9%)
*Age, years*			
Median (range)	76 (70–88)	75 (70–84)	77 (70–88)
*ECOG performance status*			
0	66 (63.5%)	22 (56.4%)	44 (67.7%)
1	38 (36.5%)	17 (43.6%)	21 (32.3%)
*Histological type*			
Intestinal	52 (50.0%)	22 (56.4%)	30 (46.2%)
Diffuse	52 (50.0%)	17 (43.6%)	35 (53.8%)
*Disease status at study entry*			
Primary	83 (79.8%)	33 (84.6%)	50 (76.9%)
Recurrence	21 (20.2%)	6 (15.4%)	15 (23.1%)
*Previous gastrectomy*			
Yes	34 (32.7%)	11 (28.2%)	23 (35.4%)
No	70 (67.3%)	28 (71.8%)	42 (64.6%)
*Metastatic sites*			
Lymph nodes	58 (55.8%)	22 (56.4%)	36 (55.4%)
Liver	35 (33.7%)	17 (43.6%)	18 (27.7%)
Peritoneum	35 (33.7%)	11 (28.2%)	24 (36.9%)
Lung	9 (8.7%)	3 (7.7%)	6 (9.2%)
Other	13 (12.5%)	5 (12.8%)	8 (12.3%)
*Ascites*			
Present	26 (25.0%)	8 (20.5%)	18 (27.7%)
Absent	78 (75.0%)	31 (79.5%)	47 (72.3%)

ECOG, Eastern Cooperative Oncology Group

### Efficacy

The median number of CapeOX treatment cycles was 6.0 in both groups. The median actual dose intensities in the original-dose and reduced-dose groups were 5732.8 mg/m^2^/week and 5437.3 mg/m^2^/week for capecitabine, and 30.1 mg/m^2^/week and 28.3 mg/m^2^/week for oxaliplatin, respectively. The median relative dose intensities in the original-dose and reduced-dose groups were 59.7% and 78.6% for capecitabine and 69.5% and 84.8% for oxaliplatin, respectively. At the time of analysis, four patients were receiving treatment, while all others had discontinued treatment. The main reason for treatment discontinuation was disease progression (62 patients, 59.6%). Treatment was discontinued due to adverse events, patient complaints, and conversion surgery in 4 (10.3%), 4 (10.3%), and 2 (5.1%) patients in the original-dose group and 5 (7.7%), 5 (7.7%), and 3 (4.6%) patients in the reduced-dose group, respectively. Adverse events delayed treatment in 29 patients (74.4%) in the original-dose group and 40 patients (61.5%) in the reduced-dose group, and reduced the oxaliplatin and capecitabine dose in 26 cases (66.7%) and 26 patients (66.7%) in the original-dose group and 24 patients (36.9%) and 29 patients (44.6%) in the reduced-dose group, respectively.

**Fig. 2:**
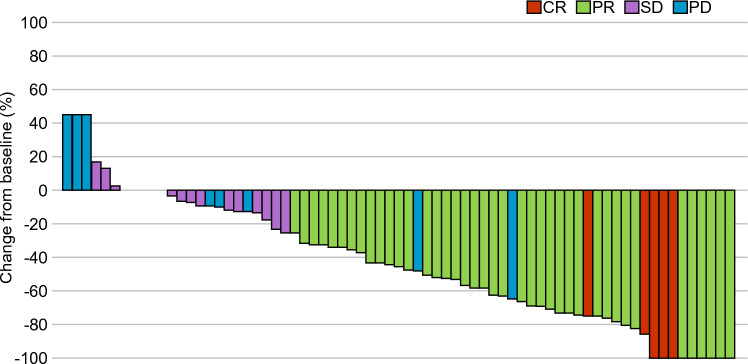
Waterfall plot showing the best overall response for each patient. CR, complete response; PR, partial response; SD, stable disease; and PD, progressive disease

The overall response rate (ORR) of patients with target lesions was 60.3% (95% CI 48.1–71.6%; complete response, *n* = 5; partial response, *n* = 39; stable disease, *n* = 15; progressive disease, *n* = 12; and not evaluable, *n* = 2). The ORR was 55.6% (95% CI 35.3–74.5%) in the original-dose group (*n* = 27) and 63.0% (95% CI 47.6–76.8%) in the reduced-dose group (*n* = 46). The disease control rate in the FAS, original-dose group, and reduced-dose group was 80.8% (95% CI 69.9–89.1%), 70.4% (95% CI 49.8–86.2%), and 87.0% (95% CI 73.7–95.0%), respectively. A waterfall plot of the best overall response for each patient is shown in Fig. 2.

**Fig. 3:**
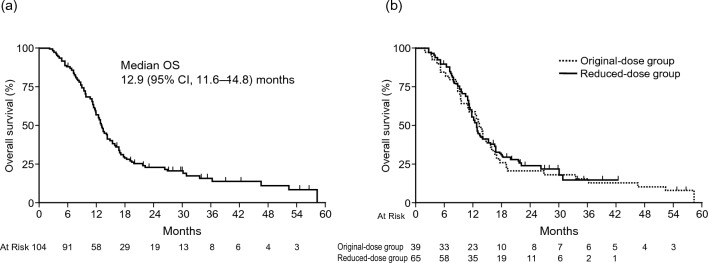
Kaplan–Meier overall survival. **a** FAS analysis and **b** analysis of the original-dose and reduced-dose groups

**Fig. 4:**
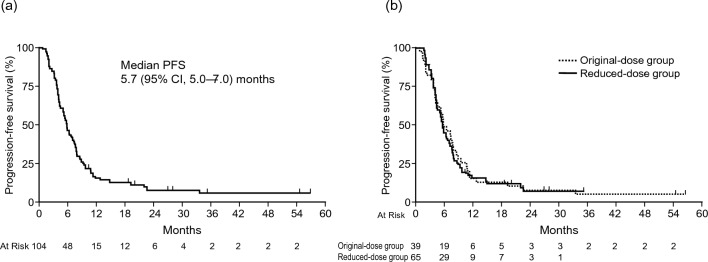
Kaplan–Meier progression-free survival. **a** FAS analysis and **b** analysis of the original-dose and reduced-dose groups

The median duration of follow-up for all patients was 40.2 months. The median OS was 12.9 (95% CI 11.6–14.8) months, and the 1-year OS rate was 56.4% (95% CI 46.2–65.3%; Fig. 3a); therefore, the null hypothesis for the primary endpoint (OS, 10 months) was rejected. The median OS in the original-dose and reduced-dose groups was 13.4 (95% CI 9.5–16.0) months and 12.8 (95% CI 11.3–15.3) months, respectively (Fig. 3b). The median PFS was 5.7 (95% CI 5.0–7.0) months, and the 1-year PFS rate was 15.4% (95% CI 9.1–23.1%; Fig. 4a). The median PFS in the original-dose and reduced-dose groups was 5.8 (95% CI 4.1–7.8) and 5.7 (95% CI 4.4–7.0) months, respectively (Fig. 4b). The median TTF was 4.3 (95% CI 3.9–5.7) months.

### Safety

All adverse events observed in the standard-dose and reduced-dose groups are summarized in Table 2. The rates of ≥ grade 3 anemia, neutropenia, and thrombocytopenia were 12.8%, 17.9%, and 12.8%, respectively, in the original-dose group and 4.6%, 13.8%, and 3.1% in the reduced-dose group. No febrile neutropenia occurred in either group. The most common ≥ grade 3 non-hematological toxicities were nausea (10.3%), anorexia (10.3%), diarrhea (7.7%), peripheral neuropathy (7.7%), and weight loss (7.7%) in the original-dose group and anorexia (12.3%), fatigue (7.7%), and peripheral neuropathy (7.7%) in the reduced-dose group. The incidence of elevated creatinine levels of any grade in the original-dose group and the reduced-dose group was 28.2% and 12.3%, respectively, and there was no ≥ grade 3 renal toxicity in any group. Heart failure did not occur in any patient.

**Table 2:** Adverse events

Event	Grade, n				All grades, n (%)	Grade ¾, n (%)
	1	2	3	4		
*Adverse events observed in the original-dose group*						
Anemia	20	13	4	1	38 (97.4%)	5 (12.8%)
Leukopenia	12	11	0	0	23 (59.0%)	0 (0%)
Neutropenia	6	9	7	0	22 (56.4%)	7 (17.9%)
Febrile Neutropenia	–	–	0	0	0 (0%)	0 (0%)
Thrombocytopenia	10	12	4	1	27 (69.2%)	5 (12.8%)
Nausea	13	6	4	0	23 (59.0%)	4 (10.3%)
Vomiting	8	0	0	0	8 (20.5%)	0 (0%)
Anorexia	10	14	4	0	28 (71.8%)	4 (10.3%)
Diarrhea	11	6	3	0	20 (51.3%)	3 (7.7%)
Fatigue	14	8	1	0	23 (59.0%)	1 (2.6%)
Hand-foot syndrome	8	7	1	0	16 (41.0%)	1 (2.6%)
Increased creatinine level	11	0	0	0	11 (28.2%)	0 (0%)
Increased AST level	26	2	0	0	28 (71.8%)	0 (0%)
Increased ALT level	16	0	1	0	17 (43.6%)	1 (2.6%)
Increased bilirubin level	2	1	1	0	4 (10.3%)	1 (2.6%)
Peripheral neuropathy	21	6	3	0	30 (76.9%)	3 (7.7%)
Weight gain	7	1	1	0	9 (23.1%)	1 (2.6%)
Weight loss	12	8	3	0	23 (59.0%)	3 (7.7%)
*Adverse events observed in the reduced-dose group*						
Anemia	39	19	3	0	61 (93.8%)	3 (4.6%)
Leukopenia	15	17	1	0	33 (50.8%)	1 (1.5%)
Neutropenia	13	17	9	0	39 (60.0%)	9 (13.8%)
Febrile Neutropenia	–	–	0	0	0 (0%)	0 (0%)
Thrombocytopenia	33	4	2	0	39 (60.0%)	2 (3.1%)
Nausea	14	8	3	0	25 (38.5%)	3 (4.6%)
Vomiting	7	0	1	0	8 (12.3%)	1 (1.5%)
Anorexia	25	13	8	0	46 (70.8%)	8 (12.3%)
Diarrhea	7	3	2	0	12 (18.5%)	2 (3.1%)
Fatigue	21	13	5	0	39 (60.0%)	5 (7.7%)
Hand-foot syndrome	21	4	1	0	26 (40.0%)	1 (1.5%)
Increased creatinine level	8	0	0	0	8 (12.3%)	0 (0%)
Increased AST level	37	3	2	0	42 (64.6%)	2 (3.1%)
Increased ALT level	29	2	0	0	31 (47.7%)	0 (0%)
Increased bilirubin level	13	2	0	0	15 (23.1%)	0 (0%)
Peripheral neuropathy	31	14	5	0	50 (76.9%)	5 (7.7%)
Weight gain	11	2	2	0	15 (23.1%)	2 (3.1%)
Weight loss	19	6	1	0	26 (40.0%)	1 (1.5%)

AST, aspartate transaminase; ALT, alanine transaminase

### Post-study treatment

Post-study treatment was performed in 75% (*n* = 75) of patients, including second-line chemotherapy in 69% (ramucirumab/taxanes 36%, taxanes 13%; ramucirumab 5%, others 15%), surgery in 5%, and radiotherapy in 1%. Third-line chemotherapy was administered to 54% of patients (nivolumab 39%, ramucirumab/taxanes 8%, taxanes, 2%; and others 5%), surgery to 1%, and radiotherapy to 1% of patients. Fourth-line, fifth-line, and sixth-line chemotherapy were administered to 14%, 7%, and 4% of patients, respectively.

## Discussion

It is conventional wisdom that older patients are frail and suffer from various comorbidities, such as low renal function. Although a combination of a platinum-based agent and a fluorouracil-based agent is regarded as an acceptable first-line chemotherapy regimen for patients with HER2-negative AGC in the sixth edition of the Japanese Gastric Cancer Treatment Guidelines, the optimal regimen for older patients has not been fully elucidated [2]. A subgroup analysis of the SPIRITS trial reported that the hazard ratio of older patients was worse than that of younger patients [6]. In contrast, a subgroup analysis of the G-SOX trial reported that S-1 and oxaliplatin (SOX) might be an effective treatment regimen in older and younger patients [14]. The WJOG8315G study was a randomized phase II study comparing SOX with S-1 monotherapy in older patients with AGC. The primary endpoint results suggested superior survival with SOX compared to that with S-1 monotherapy, along with acceptable safety; these findings were recently presented at the 2023 annual meeting of the Japanese Society of Medical Oncology [24]. These results, along with those of the present study, suggest that combination therapy with 100 mg/m^2^ of oxaliplatin and fluoropyrimidine oral anticancer agents is useful in Japanese older patients who meet the eligibility criteria.

S-1 is an oral 5-fluorouracil (5-FU) anti-tumor drug widely used in Japan due to its outpatient convenience and mild toxicity. Several meta-analyses have reported that S-1 and capecitabine could be used in mono- and combination therapy for AGC [25–27], and it may be important to select a more appropriate treatment regimen according to individual patient backgrounds by considering the different efficacy and safety profiles in each patient [28–31]. Moreover, capecitabine might be safer than S-1 in older patients, considering the distribution of tumor selectivity at high 5FU concentrations [15]. Accordingly, SOX may be replaced by CapeOX in older Japanese patients as well as overseas. However, no prospective study on CapeOX for older patients with AGC in Japan has been reported. Therefore, this multicenter prospective phase II study demonstrated that CapeOX has favorable efficacy and safety in Japanese older patients with AGC.

In our study, the median OS, PFS, and TTF among all patients were 12.9 (95% CI 11.6–14.8), 5.7 (95% CI 5.0–7.0), and 4.3 (95% CI 3.9–5.7) months, respectively, and the primary endpoint was met. In the reduced-dose group, the median OS, PFS, and TTF were 12.8 (95% CI 11.3–15.3), 5.7 (95% CI 4.4–7.0), and 4.1 (95% CI 3.7–5.7) months, respectively, and were relatively equivalent to those in the original-dose group. Moreover, the incidence of severe hematologic toxicity and elevated creatinine levels in the reduced-dose group was lower than in the original-dose group, suggesting that reduced-dose CapeOX is more suitable for older patients than original-dose CapeOX.

Several studies on CapeOX for older patients with AGC have been conducted. A phase II study reported a median OS of 9.8–10.0 months, a median PFS of 5.6–6.0 months, and an ORR of 48.9–51.2% [16, 17]. Hwang et al. reported in the first interim analysis of a multicenter phase III trial that the median OS is 11.1 months for CapeOX and 6.3 months for capecitabine monotherapy (HR 0.58; 95% CI 0.30–1.12; *P* = 0.108) and concluded that platinum-based combination chemotherapy, as compared with capecitabine monotherapy, was associated with a survival benefit in older patients with AGC [18]. In this Korean study, the initial dose of oxaliplatin was reduced to 110 mg/m^2^. Recently, Hall et al*.* reported the results of the GO2 trial, comparing three doses of CapeOX (100%, 80%, and 60%) in older and frail patients with advanced gastroesophageal cancer, and showed that reduced-dose chemotherapy provided good persistence of quality of life and low toxicity without significantly compromising cancer control [19]. These results were consistent with the current study findings that reduced-dose CapeOX was suitable for older patients with AGC. The authors also discussed the importance of avoiding toxicity-induced treatment reductions and stoppages, as they induce a negative experience for patients and detract from quality of life and cancer control.

This study has several limitations. First, the single-arm phase II study design might limit the results to being exploratory. However, to our knowledge, this is the first prospective clinical trial for CapeOX among older patients with AGC in Japan. Second, following the ATTRACTION-4 and CheckMate 649 trials, nivolumab plus chemotherapy is now the standard first-line chemotherapy [32, 33]. Although there is no direct data, this study suggests that reduced-dose CapeOX may be appropriate for older gastric cancer patients receiving CapeOX and nivolumab. Third, quality of life and patient-reported outcomes were not included as study endpoints. When administering chemotherapy to older patients, a composite outcome measure such as the Overall Treatment Utility, which combines clinical and radiological response, toxicity, adverse events, and patient-reported acceptability of treatment [34], should be adopted to individually capture the balance of benefits and harms from cancer treatments.

Older cancer patients often exhibit decreased physical function, multiple comorbidities, and geriatric syndrome, so it is important to assess individual variability when weighing the risks and benefits of treatment. However, data on vulnerable older patients is limited, as the clinical trials for the development of the standard treatment have enrolled only a subset of older patients in good health who met the eligibility criteria. The method of evaluating the physical, mental, and social functions of patients, in addition to disease evaluation, is designated comprehensive geriatric assessment (CGA). The International Society of Geriatric Oncology recommends the use of CGA when assessing older patients with cancer to detect unaddressed problems and improve their functional status and possibly their survival [35]. Notably, subset analysis in the WJOG8315G study does not suggest the superiority of SOX in the OS of patients with low Geriatric 8 (G-8) screening scores, which consist of seven items from the Mini Nutritional Assessment questionnaire and age [24]. Therefore, we will further evaluate the relationship between CGA parameters and the efficacy and safety of CapeOX in further studies.

## Conclusion

Our phase II trial results suggest that CapeOX, comprising capecitabine (750 mg/m^2^ b.i.d. on days 1–14 of each course) and oxaliplatin (100 mg/m^2^ on day 1 of each course), is effective and safe for Japanese older patients with AGC.

## Data Availability

The data that support the findings of this study are available from the corresponding author upon reasonable request. The data are not publicly available due to containing information that could compromise the privacy of research participants.

## Declarations

### Animal studies

This article does not contain any studies using animal subjects performed by any of the authors.
